# Gonadal function in males with WFS1 spectrum disorder (Wolfram syndrome)—A European cohort perspective

**DOI:** 10.1111/andr.70049

**Published:** 2025-04-29

**Authors:** Julia Rohayem, Olivia Cunningham, Denise Williams, Joachim Wistuba, Liam McCarthy, Timothy G. Barrett, Renuka P. Dias

**Affiliations:** ^1^ Department of Endocrinology and Diabetes Children's Hospital of Eastern Switzerland St. Gallen Switzerland; ^2^ Centre for Reproductive Medicine and Andrology University of Münster Münster Germany; ^3^ Department of Endocrinology and Diabetes Birmingham Children's Hospital, Birmingham Women's and Children's NHS Foundation Trust Birmingham UK; ^4^ Department of Clinical Genetics Birmingham Women's Hospital Birmingham Women's and Children's NHS Foundation Trust Birmingham UK; ^5^ Institute of Reproductive and Regenerative Biology Centre of Reproductive Medicine and Andrology, University of Münster Münster Germany; ^6^ Department of Paediatric Urology Birmingham Children's Hospital, Birmingham Women's and Children's NHS Foundation Trust Birmingham UK; ^7^ Institute of Cancer and Genomic Sciences, College of Medical and Dental Sciences University of Birmingham Birmingham UK; ^8^ Institute of Applied Health Research, College of Medical and Dental Sciences University of Birmingham Birmingham UK

**Keywords:** gonadal function, puberty, Wolfram syndrome

## Abstract

**Background:**

*WFS1* spectrum disorder, also known as Wolfram syndrome (WS) is an ultra‐rare (<1:500,000; ORPHA: 3463) monogenic (OMIM #222300) progressive neuroendocrine and neurodegenerative disorder, characterised by early‐onset insulin‐dependent diabetes, optic atrophy, central diabetes insipidus and sensi‐neuronal deafness. It is caused predominantly by bi‐allelic mutations in the *WFS1* gene and exceptionally in the *WFS2*‐gene. There is very limited published data on gonadal function in young people with WS. Expansion of the phenotype has previously included suggestions of abnormalities in puberty in adolescents with (WS) but with little detail.^1–3^

**Aim:**

To assess testicular function and pubertal progression in a cohort of adolescent and young adult patients with classical *WFS1* spectrum disorder (WS).

**Methods:**

Retrospective case notes review of national patient cohorts comprising 21 males with WS aged 16–30 years. All patients were treated in two tertiary European health care centres: in Birmingham, UK and Münster, Germany. Hormonal parameters reflecting hypothalamic–pituitary–gonadal axis function and treatment with sex hormones were assessed. In addition, the presence or absence of erectile dysfunction was explored. In a subset of men, semen data were analysed. In one young man, testicular biopsies were examined histologically using light and electron microscopy.

**Results:**

Severely delayed or arrested puberty was observed in 57% of male adolescents with WS, necessitating testosterone replacement for completion of pubertal development.

Subclinical (compensated) hypergonadotropic hypogonadism with still adequate testosterone serum concentration for age, but elevated LH/FSH was observed in 28.6% (*n* = 6). In two males, aged 19 and 16 years (9.5%), inadequately low LH/FSH and testosterone levels indicated hypogonadotropic hypogonadism.

In the subset of males with normal puberty and normal endocrine testicular function (43% of male patients), the oldest, aged 30 years had normal sperm count in semen. Another young man had oligozoospermia at age 20, but azoospermia at age 25 years. Histology of his testicular tissues evidenced structural alterations of Leydig and Sertoli cells and tubular atrophy with various stages of tubular degeneration and meiotic arrest of spermatogenesis.

**Conclusion:**

Endocrine testicular function and reproductive capacity are impaired in males with WS potentially due to premature degeneration of the testes, with 57% of adolescents developing hypogonadism with pubertal arrest.

## 1 INTRODUCTION

WFS1 spectrum disorder also known as Wolfram syndrome (WS) is an ultra‐rare (<1:500,000), progressive neurodegenerative disorder, first described by Wolfram and Wagener in 1938.^4^, ^5^ It is an autosomal recessive condition, primarily caused by mutations in the *WFS1* gene (Chr 4p16.1).^6^ *WFS1* encodes Wolframin, a transmembrane protein located in the endoplasmic reticulum (ER). It is ubiquitously expressed and has multiple functions, including as a negative regulator of the ER unfolded protein response (UPR).^7^, ^8^ Wolframin appears to function as a ‘brake’ on the UPR, without which the UPR cascade is unchecked, leading to cell death through apoptosis.

The clinical features in WS show incomplete penetrance, but optic atrophy (OA) and diabetes mellitus (DM) remain the minimum diagnostic requirements for classical WS.^9^ Classic WS was previously known as DIDMOAD for the constellation of clinical features often seen in individuals (Diabetes Insipidus, Diabetes Mellitus, Optic atrophy and Deafness). However, the phenotype is far wider than the original descriptions.^1^, ^9^, ^10^

At present, there is no cure for WS although clinical trials are currently ongoing.^11^ Treatment remains supportive and aims to reduce the significant morbidity from this condition. As medical care improves, significant numbers of young people now transition into adult care and are increasingly concerned about relationships and sexual function, as well as future fertility in clinical discussions. The current published literature on pubertal progression and gonadal function in WS is limited. Hypogonadism has been reported in small series of patients, many of whom have not completed puberty.^1^, ^2^, ^3^, ^12^

The aim of this study was to comprehensively review the gonadal function of a large cohort of young adult male patients transitioning to adult care within two highly specialised care services for *WFS1* spectrum disorder.

## 2 PATIENTS AND METHODS

### 2.1 United Kingdom

The National Health Service England Highly Specialised Service for Wolfram Syndrome (NHSE‐HSS) in Birmingham, UK, has been in place since 2012 and over the last 10 years has seen more than 60 children with a new diagnosis WS (total number of reviews within multi‐disciplinary clinic = 251). The NHSE‐HSS offers all children and young people with WS in the United Kingdom a regular (annual) clinical review with a Multi‐Disciplinary Team (MDT) clinical team. The paediatric MDT includes endocrinology, neurology, ophthalmology, urology, psychology and genetics as well as a specialist transition co‐ordinator and a family support co‐ordinator. The MDT also liaises with local services (across health, education and social care) and the patient support group Wolfram Syndrome UK (WSUK) to support families and young people in between clinic reviews as needed.

### 2.2 Germany

The Centre for reproductive Medicine and Andrology in Münster, Germany, has been the national reference centre for advice and care for adolescents with WS since 2011, with a focus on gonadal function, including reproductive capacity.

### 2.3 Ethics approval

Institutional review board approval for clinical data review was obtained from Birmingham Women's and Children's (BWC) NHS Foundation Trust (reference: CARMSID 31164) and from the Ethics Committee of the University of Münster and the Medical Board of Westfalen‐Lippe (approval number: 4 I Nie).

### 2.4 Inclusion criteria

All patients included in the retrospective data evaluation had the classical clinical WS phenotype (OA and DM before the age of 16 years) and/or confirmed biallelic mutations in *WFS1*.^13^ Only one male aged 21 did not have DM, but had genetically proven WS with known pathogenic *WFS1* mutations causing classical WS. All patients had completed growth (height velocity <2 cm/year) and/or reached the age of 16 years at the time of the study. OA was confirmed with ophthalmic examination for all patients and DM was diagnosed as per ADA criteria.^14^ Other major components of classical *WFS1* spectrum disorder were diagnosed as per standard clinical practice in each country (e.g., hearing loss through audiology assessment, bladder dysfunction through urodynamics assessment and diabetes insipidus through baseline biochemistry or with a water deprivation test^15^).

### 2.5 Definition of ‘delayed puberty’

In the present male cohort ‘pubertal delay’ was defined as testicular volumes <4 mL by age 14 years using Prader orchidometer; ‘pubertal arrest’ as inadequate progression through puberty lasting longer than 5.5 years.

The dataset included age at diagnosis and the genetic diagnosis of WS. Hormonal assessment of gonadal function, specifically LH, FSH, testosterone and inhibin B serum concentrations was undertaken. The presence of erectile dysfunction (ED) in males was noted, if documented in clinical reports.

Testicular volumes were assessed in the majority of males of the UK and German cohort by clinical assessment with Prader orchidometer (United Kingdom) and/or ultrasound (Germany).

### 2.6 Statistical analysis

Data were plotted and analysed using GraphPad Prism (V10.2.2)). A Welch's *t*‐test was performed to explore data. Give the small size and heterogeneity of the cohort, minimal downstream statistical analyses were undertaken to determine if there was a statistically significant difference between testosterone, LH, inhibin B and HbA1c between patients with ED or delayed puberty.

### 2.7 Semen analysis and testicular histology

In a subset of males from the German cohort, semen data were analysed. In one young man with azoospermia, testicular biopsies were obtained when the patient was undergoing an attempt for microsurgical testicular sperm extraction (mTESE). The procedure of mTESE has previously been described in detail.^16^Testicular tissues were evaluated histologically by both light microscopy and transmission electron microscopy (TEM). Light microscopy was performed using testicular cross sections with a thickness of 5 µm. These were stained with periodic acid Schiff (PAS) using a routine in‐house protocol.^17^ Evaluation of testicular samples by TEM EM208S (Philips, Amsterdam, the Netherlands) were prepared by fixing them in 2.5% glutaraldehyde. The samples were then post‐fixed with 1% osmiumtetroxide, subsequently dehydrated and embedded in Epon. Ultra‐thin sections (60–120 nm) were cut using a Leica Ultracut R ultramicrotome (Vienna, Austria), counterstained with 8% uranyl acetate in bi‐distilled water and incubated with lead citrate solution, as previously described.^18^

## 3 RESULTS

A total of 21 male patients with WS were included in the analysis. The median age of assessment of the cohort was 17.6 years (range 16–30.0 years). The UK cohort was primarily of non‐White ethnicity with 50% Asian (*n* = 7); 35.7% White (*n* = 5); 14.3% Black (*n* = 2). In the German cohort, all patients (100%, *n* = 7) were White. Overall, 57.1% (*n* = 12) of the cohort were White.

A total of 57.1% (*n* = 8) in the UK cohort and 28.5% (*n* = 2) in the German cohort were offspring from a consanguineous relationship. Overall, 47.6% (*n* = 10/21) of the cohort were the product of a consanguineous relationship.

In all patients, the cardinal diagnostic features of OA and non‐autoimmune DM were diagnosed before the age of 16 years, except for one male who had bi‐allelic *WFS1*‐mutations and bilateral OA with legal blindness, but no DM by age 21 years (see Table 1 for full demographic breakdown and clinical features observed in the cohort).

**TABLE 1:** Summary table of demographics and clinical features seen in the cohort.

	Males
**Median age of diagnosis (years)** ^a^	10.8 (6.9–15.7)
**Median age at most recent pubertal assessment**	17.1 (16.0–30.0)
**Median final height Standard Deviation Score**	−0.2 (−2.8 to 1.5)
**Median Body Mass Index SDS (kg/m^2^)**	1.1 (−1.5 to 2.9)
**Median age of diagnosis of diabetes mellitus (years)**	6.0 (1.5–14.5) (*note GM021 no DM at 21 years)
**Median HbA1c at final assessment (mmol/mol)**	56.3 (41–94)
**Median duration of diabetes mellitus (years)**	12.4 (4.1–16.0)
**Median age of diagnosis of optic atrophy (years)**	10.5 (4.0–17.0)

Values are given as median (range).

^a^ UK cohort only.

### 3.1 Diabetes mellitus

The median age of diagnosis of DM across the cohort was 6.0 years (range 1.5–14.5 years). One male had no evidence of dysglycaemia at age 21 years. (Patient GM021—see Table 2). The median HbA1c at final assessment was 56.3 mmol/mol (range 41–94, excluding GM021). In the UK cohort 50.0% (*n* = 7) were taking insulin via multiple daily injections (MDIs), 21.4% (*n* = 3) were using continuous subcutaneous insulin infusion (CSII) pumps and 28.6% (*n* = 4) were using a continuous glucose monitoring system (CGMS). In the German cohort of those with DM, 66.7% of males (*n* = 4) were on MDI regimens and 33.3% (*n* = 2) were using CSII.

**TABLE 2:** Detailed biochemistry for male Wolfram syndrome (WS) patients.

Patient ID	Age at assessment (years)	Mutation type (homozygous, heterozygous or compound heterozygous)	HbA1c at final assessment (mmol/mol); Normal: <38	Neurogenic bladder (Yes/No)	Mean testicular volumes (mL) and age at assessment (years)	Testosterone (nmol/L); Normal adult male reference range: >10	Luteinising hormone (U/L); Normal range 0.7–9	Follicle stimulating hormone (U/L) (normal range 1–11	Inhibin B (pmol/L); Normal >125	History of pubertal delay (Yes/No)	History of pubertal arrest (Yes/No)	Gonadal function at latest assessment	Erectile dysfunction (Yes/No)
UKM001	17	Stop	61.0	Yes	12 (15 years)	13.2	6.1	3.9	156.0	Yes	No	Eugonadal	Yes
UKM002	18	Missense/ Insertion (in‐frame)	66.0	No	8 (14 years)	22.1	6.0	4.8	220.8	No	No	Eugonadal	Yes
UKM003	17	Stop	73.0	Yes	20 (17 years)	13.4	6.1	6.1	243.6	No	No	Eugonadal	Not asked
UKM004	21	Deletion (frame shift)	42.0	No	8 (14 years)	20.6	6.5	2.9	215.2	No	No	Eugonadal	Yes
UKM005	16	Deletion (frame shift)	47.0	No	15 (15 years)	9.9	4.7	3.7	85.6	Yes	No	Eugonadal	Not asked
UKM006	16	Missense	94.0	Yes	8 (16 years)	9.6^b^	10.0	5.2	162.0	Yes	Yes	Decompensated Hypergonadotropic Hypogonadism	Not asked
UKM007^a^	19	Stop	41.0	Yes	10 (15 years)	4.8	0.7	4.6	201.7	No	Yes	Hypogonadotropic Hypogonadism	Not asked
UKM008^a^	17	Missense	50.0	Yes	8 (19 years)	21.4	15.2	17.5	64.9	Yes	Yes	Decompensated Hypergonadotropic Hypogonadism (on testosterone replacement)	Yes
UKM009^a^	16	Deletion (in‐frame)	53.0	Yes	8 (15 years)	1.4	0.9	4.5	75.5	No	No	Hypogonadotropic Hypogonadism	Not asked
UKM010	17	Stop	70.0	Yes	Not documented	9.9^b^	10.6	12.5	74.4	No	No	Decompensated Hypergonadotropic Hypogonadism	Not asked
UKM011	16	Deletion (frame shift)	63.0	Yes	Refused	n.a.	10.8	18.9	67.6	Refused assessment	Refused Assessment	Hypergonadotropic Hypogonadism unspecified (no T measurements)	Not asked
UKM012	19	Stop	81.0	Yes	15 (17 years)	10.2	17.6	11.7	39.7	No	No	Compensated Hypergonadotropic Hypogonadism	Not asked
UKM013	17	Stop	57.0	Yes	4 (15 years)	10.6	18.1	33.2	35.5	Yes	No	Compensated Hypergonadotropic Hypogonadism	Not asked
UKM014	17	Stop	83.6	Yes	Not documented	13.9	5.5	3.3	133.0	No	No	Eugonadal	Not asked
GM015^a^	22	Stop (hom)	56.3	Yes	3 (22 years)	4.2^b^	15.6	35.3	<9	No	Yes	Decompensated Hypergonadotropic Hypogonadism	Yes
GM016^a^	20	Stop (hom)	57.4	Yes	4 (20 years)	7.6^b^	38.7	106	<9	No	Yes	Decompensated Hypergonadotropic Hypogonadism	Yes
GM017^a^, ^d^	25	Missense (hom)^d^	51.9	Yes	12 (25 years)	7.2^b^	4.0	14.9	38	No	Yes	Decompensated Hypergonadotropic Hypogonadism	Yes
GM018^a^	26	Stop (hom)	50.8	Yes	4 (26 years)	2.7^b^	0.7	6.9	<9	No	Yes	Decompensated Hypergonadotropic Hypogonadism	Yes
GM019	30	Missense (comp het)	49.7	Yes, mild	30 (30 years)	31.1	3.6	4.1	251.1	No	No	Eugonadal	No
GM020	16	Missense (comp het)	44.3	No	20 (16 years)	12	1.3	6.6	144.9	No	No	Eugonadal	No
GM021^c^	21	n.a.	35.5	Yes	24 (21 years)	12.8	3.0	3.9	n.a.	No	No	Eugonadal	Not asked

Abbreviations: comp.het., compound heterozygous; het., heterozygous; hom., homozygous; n.a., not available.

^a^ Testosterone replacement given.

^b^ Decompensated hypergonadotrophic hypogonadism.

^c^ Had not developed diabetes mellitus at assessment.

^d^ Patient who underwent mTESE.

### 3.2 Optic atrophy, deafness, central diabetes insipidus and neurogenic bladder dysfunction (UK cohort)

The median age at diagnosis of OA was 10.5 years (range 4.0–17.0 years); 35.7% (*n* = 5/14) were registered as partially sighted and 7.2% were registered blind (*n* = 1/14) at the time of assessment.

A total of 78.6% (*n* = 11/14) had hearing issues of which 28.6% (*n* = 4/14) required hearing aids. A total of 42.8% (*n* = 9/21) had cranial diabetes insipidus at their most recent assessment; 71.4% (*n* = 10/14) had a neurogenic bladder, of which 21.4% (*n* = 3/14) required intermittent or permanent catheterisation.

### 3.3 Endocrine and spermatogenic testicular function in males with Wolfram syndrome

Detailed results regarding reproductive hormones (testosterone, LH, Follicle Stimulating Hormone (FSH) and inhibin B) of the male WS patients at the most recent assessment are shown in Table 2. Summary biochemistry is given in Table 3. None of the patients had clinical or ultrasonographic features of crypto‐orchidism, hypospadias or varicocoele.

**TABLE 3:** Summary table of biochemistry of males with and without erectile dysfunction or delayed puberty (see also Table 1 for individual data).

	Testosterone (nmol/L); normal adult male range: 7–27	Luteinising hormone (IU/L); normal adult male range: 0.7–9.1	Follicle stimulating hormone (IU/L); normal adult male range 1–11 IU/L	Inhibin B (ng/L) normal adult male reference: >125	HbA1c (mmol/mol); normal: <38
All males	10.6 (1.4–22.1)	6.3 (0.7–18)	5 (2.9–33.2)	109.3 (35.5–33.2)	62 (41–94)
Males with delayed or arrested puberty (*n* = 11)	13.3 (9.6–21.1)	6.3 (4.7–18.1)	5.0 (2.9–33.2)	159.0 (35.5–243.5)	59 (42–94)
Males without delayed puberty (*n* = 10)	9.9 (1.4–13.9)	8.1 (0.7–17.6)	8.2 (3.3–33.2)	75.0 (39.7–201.7)	63 (41–81)
Eugonadal	16.5 (9.9.‐31.1)	4.8 (1.3–6.5)	4.4 (2.9–6.6)	181.3 (85.6–251.1)	55.7
Subclinical hypergonadotropic hypogonadism^b^	10.4	17.85	22.45	37.6	69
Decompensated hypergonadotrophic hypogonadism^a^	6.9 (2.7–9.9)	13.5 (0.7–38.7)	28.3 (5.2–35.2)	58.6 (<9–162)	61.5 (50–94)
Hypogonadotrophic hypogonadism^b^	3.1	0.8	4.55	138.6	47

**V**alues are given as median (range).

^a^ Patient on testosterone replacement removed due to skewing of data.

^b^ Only two patients.

A total of 42.8% (9/21) of the patients were eugonadal, 9.5% (2/21) had subclinical (compensated) hypergonadotrophic hypogonadism and 33.3% (7/21) had decompensated hypergonadotrophic hypogonadism. A total of 9.5% (2/21) had hypogonadotrophic hypogonadism, and one (4.8%) had hypergonadotropic hypogonadism unspecified (no serum testosterone measurements available; see Table 2).

**FIGURE 1:**
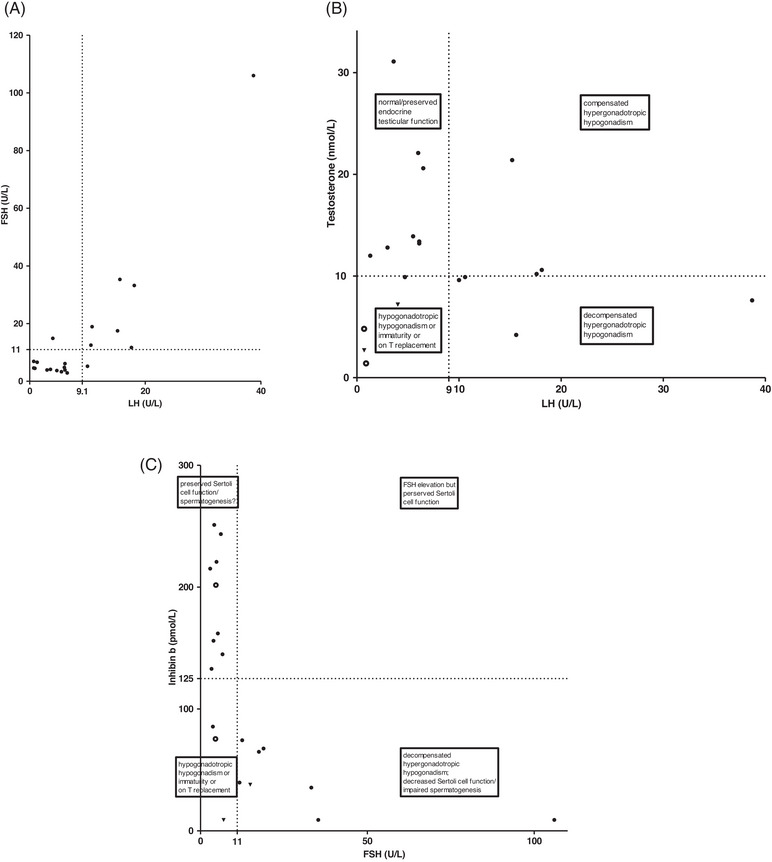
Hypothalamic–pituitary–gonadal axis function in male Wolfram syndrome (WS) patients. Graph showing (A) LH (U/L) versus FSH (IU/L), (B) LH (U/L) versus testosterone (nmol/L), and (C) FSH (U/L) versus inhibin B (pmol/L) in 20 male WS patients. In figure B and C, solid triangles indicate that the patient is on replacement with testosterone, open circles indicate the patient has hypogonadotropic hypogonadism.

Nine of the 21 males with WS (42.8%) had preserved endocrine testicular function at the time of the last assessment, while eight showed evidence of elevated Luteinisimg Hormone (LH) (>10 IU/L). Two of those, had testosterone levels >10 nmol/L, despite enhanced LH stimulation, indicating compensated (subclinical) hypergonadotropic hypergonadism, six had decompensated hypergonadotropic hypogonadism, that is, testosterone concentration below the normal adult range, despite increased LH serum levels and were replaced with testosterone thereafter (Table 2 and Figure 1B).

In 7/21 WS males (UKM001, UKM002, UKM 004, UKM 005, UKM016, UKM012 and UKM014), higher serum LH compared with FSH levels were demonstrated in the compensated state of hypogonadism (see Table 2 and Figure 1A).

Two males had partially suppressed serum LH levels, as testosterone replacement had been implemented previously due to hypergonadotropic gonadal failure (patient G017 and G018; Figure 1B).

Fifty‐five percent (11/20) of the male WS patients had inhibin B levels <125 pmol/L, thus below the normal adult range, indicative of altered Sertoli cell function and/or impaired spermatogenesis.

Figure 1C shows the relationship between inhibin B and FSH levels. There appears to be a tendency for inhibin B to be lower, the higher the FSH levels are (*r* ^2^ = 0.21). When inhibin B is assessed against age (excluding patients with sub‐optimal inhibin B levels), there is a weak correlation (*r* ^2^ = 0.13, *p* = 0.38; Supporting Information Figure 1), which is expected.^19^

Two males aged 19 years and 16 years (16.7%; patient UKM007 and UKMM009 Table 2; Figure 1B) showed evidence of hypogonadotropic hypogonadism, with inadequately low levels of serum LH (<1 IU/L), and FSH (<5 IU/L) and testosterone (<7 nmol/L), indicating either extreme constitutional delay of growth and puberty (most likely) or hypogonadotropic hypogonadism.

Semen analysis was not possible in many of the adolescents, due to disrupted puberty or psychological issues. It was only performed in two young men (GM017, GM19) with WS during adulthood. In one, semen was repeatedly analysed during adulthood, due to his wish for paternity. However this patient had required testosterone replacement for pubertal arrest, which suppressed his LH and FSH levels. Therefore, substitution with testosterone undecanoate was switched to testosterone gel; nevertheless, gonadotropin suppression and azoospermia persisted. Thereafter, hCG and rFSH were used for more 24 months, but without success regarding induction of spermatogenesis. In a male aged 30 years with biochemical evidence of normal gonadal function (GM019; normal LH, FSH levels and normal adult serum testosterone concentrations), semen analysis showed normozoospermia (GM 019). In the other  young man with compensated hypergonadotropic hypogonadism (GM017) at age 20 years, semen analysis showed the presence of few elongated spermatids (oligozoospermia), although he had intermittently been replaced with testosterone enanthate (TE). However, 5 years later, at age 25, when this young man planned to father a child, high FSH (14.9 U/L), but inadequately normal LH (presumably due to previous testosterone application; 4 U/L) for low testosterone (7.2 U/L) indicated hypergonadotropic hypogonadism. Azoospermia was diagnosed, and persisted even after the patient had then adequately paused TE for more than 4 months before semen analysis. Microsurgical testicular sperm retrieval (mTESE) was then performed after pre‐treatment with hCG over 4 months in the attempt to increase intratesticular testosterone, which is necessary for spermatogenesis. However, mTESE was not successful in retrieving testicular spermatozoa.

### 3.4 Histology of testicular tissues by light and transmission electron microscopy

**FIGURE 2:**
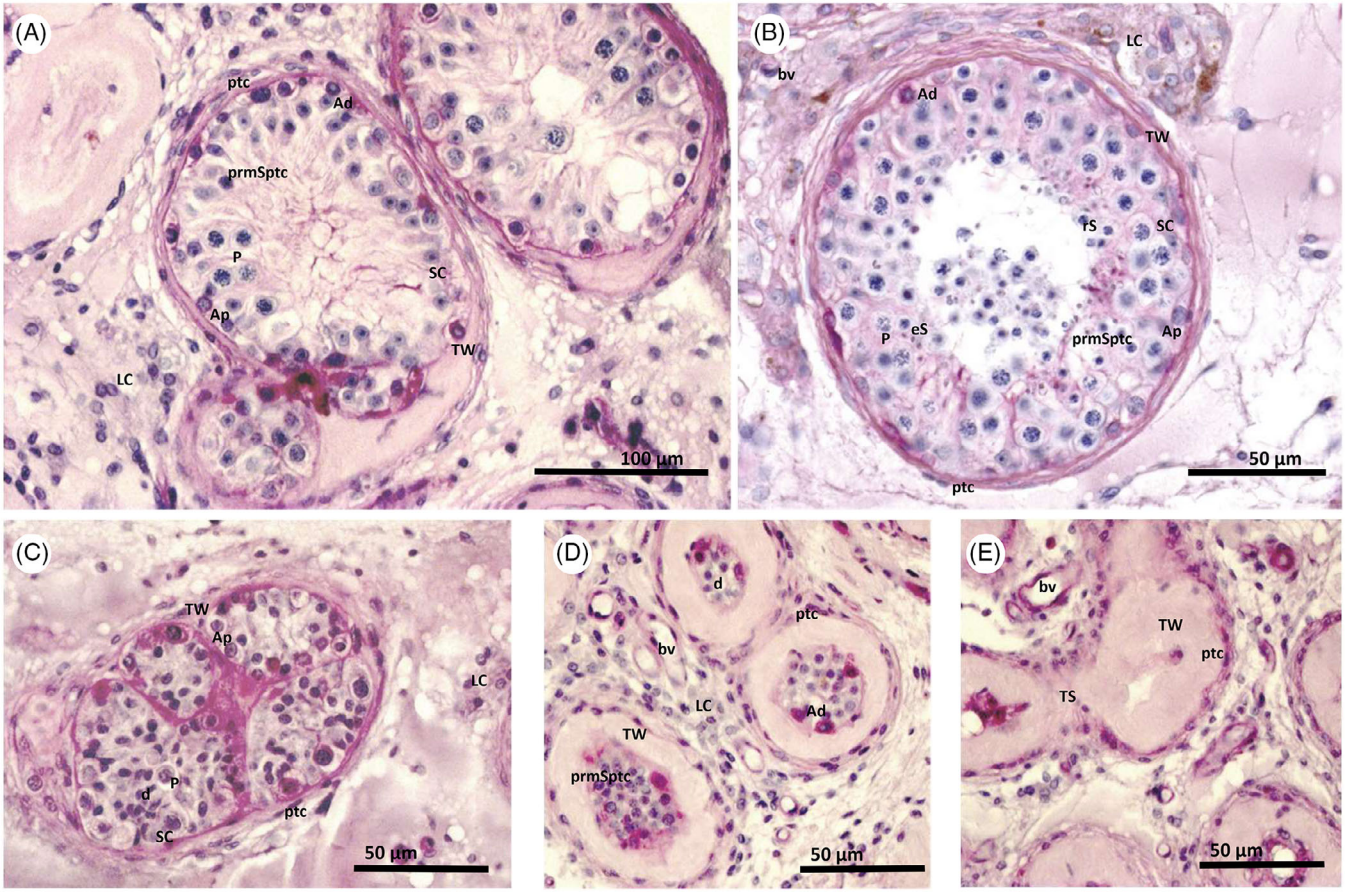
Light microscopy of testicular tissue stained with periodic acid Schiff (PAS) of an azoospermic 25‐year‐old men with Wolfram syndrome who underwent microsurgical testicular sperm extraction (mTESE; micrographs from the Centre for Reproductive Medicine and Andrology, University of Münster, Germany). Testicular tissues (A, C, D, E) show an ongoing degenerative processes in comparison to normal spermatogenesis (B). Spermatogenic development is truncated at the meiotic stage within the most differentiated tubular phenotype (A). These tubules reveal a mostly normal organisation of the germinal epithelium, however, postmeiotic haploid cells as round (rS) and elongated spermatids (eS) are no longer present. The distribution of blood vessels (bv) Leydig cells (LC), Sertoli cells (SC), peritubular myoid cells (ptc), A dark (Ad) and A pale (Ap) spermatogonia and the premeiotic spermatocytes (prm Sptc) appear normal, the abundance of the meiotic pachytene spermatocytes (P), however seemed reduced. Various stages of tubular degeneration are observed in the testicular tissues: Tubules reduced in size with disorganised epithelium, exhibiting numerous dying germ cells, aside those still differentiating (C), tubules with hyalinised tubular walls (TW) in which the cellular arrangements are totally gone (D) and tubular shadows (TS) in which all intratubular cells are absent, which corresponds to the final stage of tubular degeneration.

Light microscopy of the testicular tissues of this 25 year old man (GM017) evidenced tubular atrophy with various stages of tubular degeneration. There were tubules reduced in size with disorganised epithelium, exhibiting numerous dying germ cells, signs of still differentiating tubules, and tubules with hyalinised walls, in which the physiologic cellular arrangements were totally absent. In addition, tubular shadows corresponding to a final stage of tubular degeneration were seen; in these, all intratubular cells were absent. In a few tubules, still displaying spermatogenic development, meiotic arrest of spermatogenesis was evidenced, with absence of elongated spermatids (Figure 2).

**FIGURE 3:**
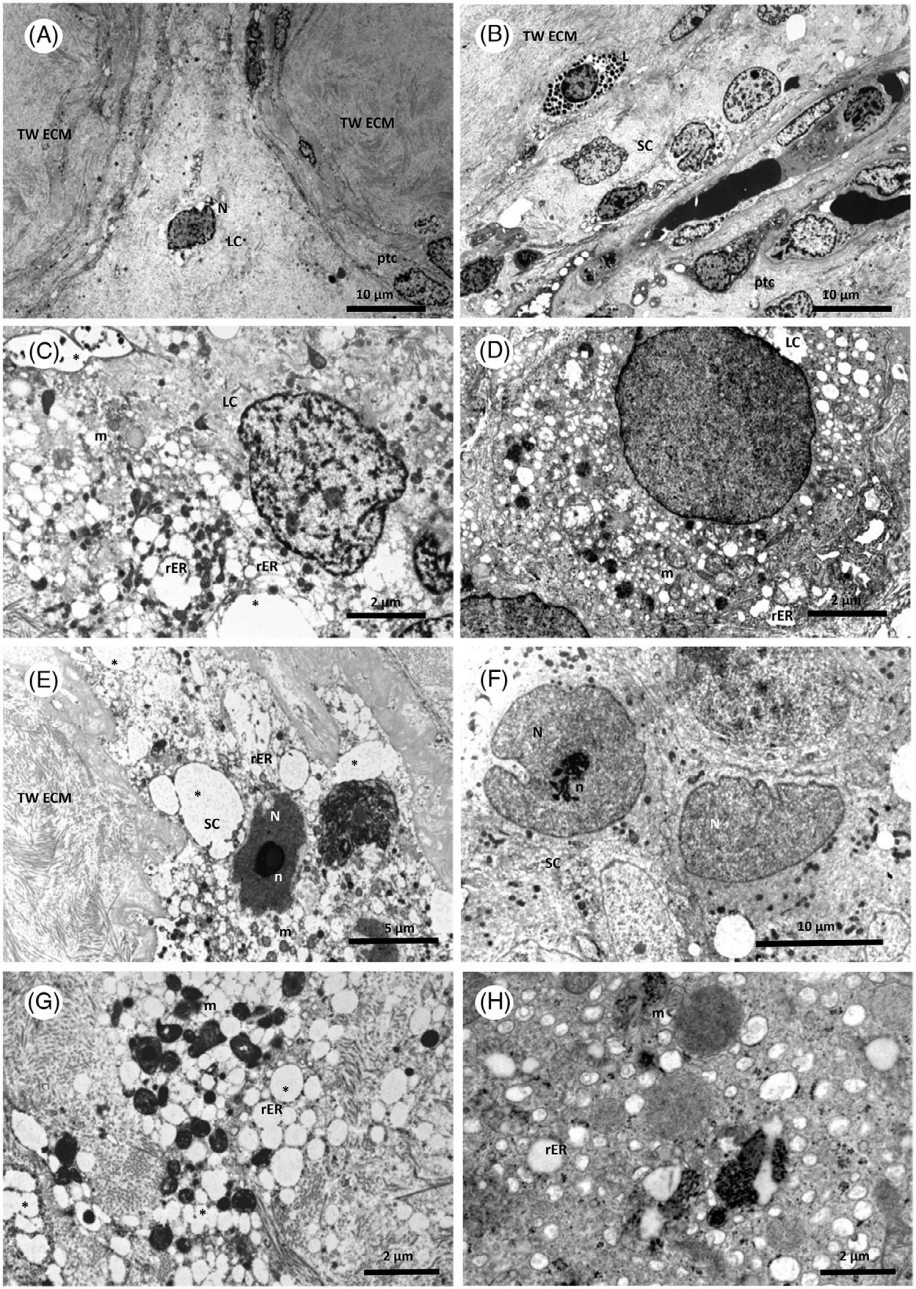
Transmission electron microscopy of testicular tissues from the same patient (micrographs from the Institute for Pathology, Münster, University of Münster, Germany). Testicular tissues (A, B, C, E, G) of the patient in comparison to control tissue of a men without Wolfram syndrome (D, F, H) are marked by big amounts of extracellular matrix material from the walls of degenerated tubules (TW ECM). Peritubular myoid cells (ptc) are still existent. Somatic cells show signs of degeneration (here likely a Leydig cell (LC) nucleus (N), (A)). Many testicular cells present with numerous electron dense lysosomes (L) and nuclei showing a speckled pattern of chromatin distribution in regions with massive accumulations of the extracellular matrix, forming the hyalinised tubular walls (TW ECM) (B). The comparison of a LC from the patient (C) with a normal one (D) also shows degenerative signs. The chromatin distribution in the nucleus (N) is altered and irregular, the mitochondria (m) appear enlarged. Vesicles from the rough endoplasmic reticulum (rER) are increased in diameter and number. When comparing a Sertoli cell from the patient (E) to a normal Sertoli cell (F), the structure of the nucleus (N) and the nucleolus (n) appear more electron dense, mitochondria appear enlarged and more electron light and the rER vesicles are substantially enlarged. These differences are also shown in more detail in (G and H), with the cytoplasmic structure and the subcellular organelles from the somatic cells of the patient (G) versus those from normal tissue (H) at a higher magnification.

TEM of testicular samples of the same man showed degeneration of Leydig cells. This was evidenced by altered and irregular chromatin distribution in the nucleus, enlarged mitochondria and increased size and number of vesicles from the rough endoplasmic reticulum (rER). Sertoli cells from the patient showed an altered (more electron dense) structure of the nucleus and the nucleolus, enlarged mitochondria, and substantially enlarged rER vesicles (Figure 3).

### 3.5 Erectile function and bladder function

A query of ED was documented in 11 patients (four UK males and seven German males), and 88.9% (*n* = 8/11) patients reported ED, even after testosterone had been adequately replaced. Six of these patients (86%) had delayed puberty.^1^ No formal assessment of ED was undertaken.

Eleven male UK patients had neurogenic bladder (78.6%) with only one of these (9.1%) requiring clean intermittent catheterisation. Six of seven males (86%) of the German cohort had bladder issues, with two having more severe problems with neurogenic bladder dysfunction (with residual urine volumes in the upper normal range) and one of the German patients required catheterisation at age 35 years.

There was no association between ED and treatment for neurogenic bladder (e.g., intermittent or permanent catheterisation) in the male UK cohort, but the two males in the German cohort who asked for medical treatment for ED also had the most severe problems with emptying of the bladder.

Forty percent (*n* = 6/15) male patients with a documented relationship status were in a romantic or intimate relationship.

A Welch's *t*‐test was performed to determine if there was a statistically significant difference between testosterone, LH, inhibin B and HbA1c between patients with ED or delayed puberty. No significant differences were found, see Table 3. There was also no association between inhibin B levels and testosterone levels.

### 3.6 Genotype and‐phenotype in male WS patients

Data on the results of *WFS1*‐mutation analysis are detailed in Table 4.

**TABLE 4:** Full genotype data of the male Wolfram syndrome (WS) patient cohort.

Patient ID	Nucleotide change	Amino acid change	Mutation type	Pathogenicity
UKM001	allele 1 c.334C>T allele 2 c.334C>T	p.Gln112X	stop (hom.)	Pathogenic
UKM002	allele 1 c.937C>T (de novo) allele 2 c.‐ 170914dup TGCCCC	p.His313Tyr in‐frame	missense (het.) Insertion (in‐frame) (het.)	Pathogenic/likely pathogenic
UKM003	allele 1 c.334C>T allele 2 c.334C>T	p.Gln112X	stop (hom.)	Pathogenic
UKM004	allele 1 c.2648_2651delTCTT; allele 2 c.2648_2651delTCTT	p.PheSerf*68;	deletion (frame shift) (hom.)	Pathogenic/likely pathogenic
UKM005	allele 1 c.2643_2644del allele 2 c.2643_2644del	p.Phe883Leufs*56	deletion (frame shift) (hom.)	Pathogenic/likely pathogenic
UKM006	allele 1 c.2654C>T allele 2 c.2654C>T	p.Pro885Leu	missense (hom.)	Pathogenic/likely pathogenic
UKM007	allele 1 c.2099G>A allele 2 c.2099G>A	p.Trp700X	stop (hom.)	Pathogenic
UKM008	allele 1 c.548T>C allele 2 c.505G>A	p.Glu169Lys p.Met183Thr	missense (comp.het.)	Variant of Uncertain Significance Pathogenic
UKM009	allele 1 c.1525_1539del15 allele 2 1 c.1525_1539del15	p.Val509_Tyr513del	deletion (in‐frame) (hom.)	Pathogenic/likely pathogenic
UKM010	allele 1 c.334C>T allele 2 c.334C>T	p.Gln112X	stop (hom.)	Pathogenic
UKM011	allele 1 c.1230_1233 delCTCT allele 2 c.1356_1371 del GCCCTACACGCGCAGG	p.Val412Serfs p.Glu452Argfs	deletion (frame shift) (comp het.)	Pathogenic Pathogenic
UKM012	allele 1 c.334C>T allele 2 c.334C>T	p.Gln112X	stop (hom.)	Pathogenic
UKM013	allele 1 c.2099G>A allele 2 c.2099G>A	p.Trp700X	stop (hom.)	Pathogenic
UKM014	allele 1 c.334C>T allele 2 c.334C>T	p.Gln112X	stop (hom.)	Pathogenic
GM015	allele 1 c.1096C>T allele 2 c.1096C>T	p.Glu266X	stop (hom.)	Pathogenic
GM016	allele 1 c.1096C>T allele 2 c.1096C>T	p.Glu366X	stop (hom.)	Pathogenic
GM017	allele 1 c.629C>T allele 2 c.629C>T	p.Pro210Leu	missense (hom.)	Variant of unknown significance (likely pathogenic)
GM018	allele 1 c.1619G>A allele 2 c.1619G>A	p.Trp480X	stop (hom.)	Pathogenic/likely pathogenic
GM019	allele 1 c.1619G>A allele 2 c.2642T>G	p.Trp540* p.Phe881Cys	missense (comp.het.)	Pathogenic/likely pathogenic
GM020	allele 1 c.1672C>T allele 2 c.2104 G>T	p.Arg558Cys p.Gly702Cys	missense (comp.het.)	Variant of Uncertain Significance Pathogenic/likely pathogenic
GM021	n.a.	n.a.	n.a.	Unknown

**Abbreviations**: comp.het., compound heterozygous; het., heterozygous; hom., homozygous; n.a., not available; PVS1, null variant.^28^

Fifty percent (10/20) of the male cohort with full genotyping data had homozygous stop mutations in their *WFS1* genes, and 20% (4/20) had deletions (of those 75% had homozygous and 25% compound heterozygous deletions). Twenty‐five percent had missense mutations (of which 40% were homozygous and 60% were compound heterozygous) and 5% (1/20) were heterozygous for a missense and an in‐frame insertion.

The most severe phenotype was observed in patients GM015, GM016 and GM018, who all had homozygous stop mutations, with undetectable inhibin B serum concentrations from adolescence onwards, early pubertal arrest, necessitating testosterone supplementation together with early‐onset ED and compensated neurogenic bladder dysfunction.

However, in all the others, no correlation could be established regarding the *WFS1* genotype and the severity of gonadal dysfunction.

### 3.7 Psychological aspects

Two out of 21 (9.5%) of the male patients had anxiety or depression requiring medication. None had had psychiatric intervention or assessment. Two out of 21 (9.5%) had additional learning needs.

## 4 DISCUSSION

This is the first paper describing in detail the gonadal phenotype of a large bi‐national cohort of young adult male patients with WS.

We observed that delayed or arrested puberty is common in male adolescents with WS, affecting around 50% of patients. We found that the pubertal disorder in males is mostly due to primary testicular dysfunction, as reflected by hypergonadotropic hypogonadism, and rarely due to central GnRH or gonadotropin deficiency (hypogonadotropic hypogonadism). It is likely that the two patients seen with apparent hypogonadotrophic hypogonadism represent extreme constitutional delay of growth and puberty.

Another important result of the present study is that ED is very common at a young age in males with WS, and not resolved by testosterone replacement. ED was only partially responsive to PDE5 inhibitors. The prevalence of ED is striking, given the general population prevalence is approximately 8% in young men under 30 years.^20^ We speculate that ED is the result of the neuro‐endocrine degenerative process occurring in WS.

We observed a high proportion of reduced serum inhibin B levels in males from adolescence onwards. The majority of these patients had evidence of subclinical or manifest hypogonadism. Lower levels have been shown to be a marker of impaired Sertoli cell function and altered spermatogenesis, although the range in normospermic males is highly variable.^21^

We also observed an unusual ratio of LH:FSH in several males, who were not on testosterone replacement, with higher LH serum levels than FSH‐levels. This is notable because in adolescent hypogonadism of other causes, spermatogenic dysfunction (as indicated by elevated FSH and low inhibin B) always precedes endocrine decompensation of the testes. Our observation of high LH serum levels in male adolescents with WS is suggestive of an unusually severe degree of early Leydig cell dysfunction. This is supported by our histologic findings, in which severe degeneration of Leydig cells was evidenced, in parallel to degeneration of spermatogenic tubules in a young men with WS.

Our semen data confirm that spermatogenesis is highly disturbed in young men with WS.

The phenotypic variability with respect to gonadal function encountered in young males with WS, ranging from eugonadal function to subclinical hypergonadotrophic hypogonadism with normal testosterone levels, and to hypogonadotrophic hypogonadism associated with low testosterone fits in with mice models previously reported.^22^ However the rodent model for WS is not an ideal for understanding human fertility.

It is unclear if diabetes control has an impact on gonadal function in WS. In patients with type 1 diabetes mellitus (T1D), fertility issues are documented in adult males with lower rates of fertility, however, sperm concentrations, sperm morphology and motility in men affected by T1D are not always altered, compared with controls.^23^ Effects of repetitive hyperglycaemic states and increased glucose variability on gonadal function have not been investigated in Wolfram patients, yet, and were not within the focus of the present study.

Fertility data in WS are limited. A 2012 study of two Iranian families identified two males with classic WS—both phenotypically and genotypically, who had unaffected offspring. The offspring of these two males were heterozygous carriers for the mutations.^24^ These males are the only documented to have successfully fathered children. There have also been six reported successful pregnancies in women with WS.^25^, ^26^ None of our young adult cohort had had offspring at the time of assessment.

Anotherimportant finding of our study is that there was a high level of bladder dysfunction, which has not been reported before in the literature. In one study, the prevalence of renal or bladder problems by the age of 18 years was reported to be 11.4%, while other reports suggest that up to 50% of patients develop bladder dysfunction by the age of 20 years.^10^, ^27^ Assessment of bladder dysfunction is a clear need in young men with WFS1 Spectrum Disorder.

### 4.1 Strengths

This is the largest cohort of young adult males with WS to be reported across two centres in Europe and the first report of inhibin B levels and semen analysis.

### 4.2 Limitations

A limitation of the present study is that longitudinal data for inhibin B and gonadotrophins as well as pubertal progression was not available. In the UK cohort, very few patients had testicular volumes assessed at the time of transition—COVID‐19 meant several appointments remained virtual over the time of transfer to adult services. ED was assessed without using validated questionnaires.

We also observed a high level of psychological issues across the cohort. This suggests that there is a high level of vulnerability in terms of depression and anxiety during adolescence.

We therefore recommend that the onset and progression of puberty should be closely monitored in adolescent patients with WS. Documentation of pubertal Tanner stages in conjunction with regular measurement of serum LH, FSH, testosterone/oestradiol and inhibin B levels are mandatory to allow early diagnosis of pubertal delay or arrest and timely initiation of hormone replacement therapy. Assessing sexual function during adolescence is particularly important in young men with WS, as it may allow adjunctive measures to be initiated to preserve fertility and/or improve erectile function.

## 5 CONCLUSIONS

Endocrine testicular function and reproductive capacity can be impaired (most often moderately) in males with WS due to premature degeneration of the testes.

Monitoring of gonadal function appears to be essential from early adolescence onwards in young men with WS to allow timely fertility preservation measures to be taken before endocrine testicular function ceases and lifelong testosterone replacement becomes necessary. Clarification of ED is also important to allow for additional therapeutic measures.

## AUTHOR CONTRIBUTIONS

Renuka P. Dias, J. Rohayem, L. McCarthy, T. G. Barrett contributed clinical data. Joachim Wistuba undertook imaging and analysis of the testicular tissue. Renuka P. Dias and J. Rohayem analysed data and co‐wrote the manuscript. Renuka P. Dias conceived article and had final responsibility for the decision to submit for publication.

## CONFLICT OF INTEREST STATEMENT

Renuka P. Dias has received honoraria from Sanofi for participation in advisory board on Teplizumab in Type 1 Diabetes. Other authors declare no conflicts of interest.

## Supporting information



Supporting Information

## Data Availability

The data underlying this article are available in the article and in its online Supporting Information.

## References

[1] Rohayem J , Ehlers C , Wiedemann B , et al. Diabetes and neurodegeneration in Wolfram syndrome: a multicenter study of phenotype and genotype. Diabetes Care. 2011;34(7):1503‐1510. doi:10.2337/dc10-1937 21602428 PMC3120194

[2] Bueno GE , Ruiz‐Castañeda D , Martínez JR , Muñoz MR , Alascio PC . Natural history and clinical characteristics of 50 patients with Wolfram syndrome. Endocrine. 2018;61(3):440‐446. doi:10.1007/s12020-018-1608-2 29728875

[3] Frontino G , Di Tonno R , Stancampiano MR , et al. Paediatric Wolfram syndrome Type 1: should gonadal dysfunction be part of the diagnostic criteria?. Front Endocrinol (Lausanne). 2023;14:1155644. doi:10.3389/fendo.2023.1155644 37383390 PMC10294676

[4] Wolfram DJ , Wagener HP . Diabetes mellitus and simple optic atrophy among siblings: report on four cases. Mayo Clin Proc. 1938;13:715‐718.

[5] Barrett TG , Bundey SE , Macleod AF . Neurodegeneration and diabetes: UK nationwide study of Wolfram (DIDMOAD) syndrome. Lancet. 1995;346(8988):1458‐1463. doi:10.1016/s0140-6736(95)92473-6 7490992

[6] Inoue H , Tanizawa Y , Wasson J , et al. A gene encoding a transmembrane protein is mutated in patients with diabetes mellitus and optic atrophy (Wolfram syndrome). Nat Genet. 1998;20(2):143‐148. doi:10.1038/2441 9771706

[7] Takei D , Ishihara H , Yamaguchi S , et al. WFS1 protein modulates the free Ca(2+) concentration in the endoplasmic reticulum. FEBS Lett. 2006;580(24):5635‐5640. doi:10.1016/j.febslet.2006.09.007 16989814

[8] Zatyka M , Da Silva Xavier G , Bellomo EA , et al. Sarco(endo)plasmic reticulum ATPase is a molecular partner of Wolfram syndrome 1 protein, which negatively regulates its expression. Hum Mol Genet. 2015;24(3):814‐827. doi:10.1093/hmg/ddu499 25274773 PMC4291252

[9] Barrett T , Tranebjærg L , Gupta R . WFS1 Spectrum Disorder. [Updated 2022 Dec 1]. In: Adam MP , Feldman J , Mirzaa GM , eds. GeneReviews® [Internet]. Seattle (WA): University of Washington, Seattle; 1993–2025. Available from: https://www.ncbi.nlm.nih.gov/books/NBK4144/ 20301750

[10] de Heredia ML , Clèries R , Nunes V . Genotypic classification of patients with Wolfram syndrome: insights into the natural history of the disease and correlation with phenotype. Genet Med. 2013;15(7):497‐506. doi:10.1038/gim.2012.180 23429432

[11] Dias RP , Brock K , Hu K , et al. Sodium valproate, a potential repurposed treatment for the neurodegeneration in Wolfram syndrome (TREATWOLFRAM): trial protocol for a pivotal multicentre, randomised double‐blind controlled trial. BMJ Open. 2025;15(2):e091495. doi:10.1136/bmjopen-2024-091495 PMC11865774 40010822

[12] Das L , Rai A , Mavuduru R , et al. Wolfram syndrome: clinical and genetic profiling of a cohort from a tertiary care centre with characterization of the primary gonadal failure. Endocrine. 2020;69(2):420‐429. doi:10.1007/s12020-020-02320-6 32350710

[13] Barrett T , Tranebjærg L , Gupta R , et al. WFS1 Spectrum Disorder . 2023. Accessed February 2, 2009. https://www.ncbi.nlm.nih.gov/books/NBK4144/

[14] Committee ADAPP . 2. Diagnosis and classification of diabetes: standards of care in diabetes—2024. Diabetes Care. 2023;47(suppl 1):S20‐S42. doi:10.2337/dc24-S002 PMC10725812 38078589

[15] Gubbi S , Hannah‐Shmouni F , Koch CA , Verbalis JG . Diagnostic Testing for Diabetes Insipidus. Endotext [Internet]. MDText.com, Inc; 2022. Accessed January 18. https://www.ncbi.nlm.nih.gov/books/NBK537591/

[16] Rohayem J , Haffner D , Cremers JF , et al. Testicular function in males with infantile nephropathic cystinosis. Hum Reprod. 2021;36(5):1191‐1204. doi:10.1093/humrep/deab030 33822926 PMC8058591

[17] Brinkworth MH , Weinbauer GF , Schlatt S , Nieschlag E . Identification of male germ cells undergoing apoptosis in adult rats. J Reprod Fertil. 1995;105(1):25‐33. doi:10.1530/jrf.0.1050025 7490711

[18] Reynolds ES . The use of lead citrate at high pH as an electron‐opaque stain in electron microscopy. J Cell Biol. 1963;17(1):208‐212. doi:10.1083/jcb.17.1.208 13986422 PMC2106263

[19] Kelsey TW , Miles A , Mitchell RT , Anderson RA , Wallace WHB . A normative model of serum inhibin B in young males. PLoS One. 2016;11(4):e0153843. doi:10.1371/journal.pone.0153843 27077369 PMC4831823

[20] Rosen RC , Fisher WA , Eardley I , Niederberger C , Nadel A , Sand M . The multinational Men's Attitudes to Life Events and Sexuality (MALES) study: I. Prevalence of erectile dysfunction and related health concerns in the general population. Curr Med Res Opin. 2004;20(5):607‐617. doi:10.1185/030079904125003467 15171225

[21] Jensen TK , Andersson AM , Hjollund NH , et al. Inhibin B as a serum marker of spermatogenesis: correlation to differences in sperm concentration and follicle‐stimulating hormone levels. A study of 349 Danish men. J Clin Endocrinol Metab. 1997;82(12):4059‐4063. doi:10.1210/jcem.82.12.4456 9398713

[22] Noormets K , Kõks S , Muldmaa M , Mauring L , Vasar E , Tillmann V . Sex differences in the development of diabetes in mice with deleted wolframin (Wfs1) gene. Exp Clin Endocrinol Diabetes. 2011;119(5):271‐275. doi:10.1055/s-0030-1265163 21031341

[23] Facondo P , Di Lodovico E , Delbarba A , et al. The impact of diabetes mellitus type 1 on male fertility: systematic review and meta‐analysis. Andrology. 2022;10(3):426‐440. doi:10.1111/andr.13140 34904793

[24] Haghighi A , Haghighi A , Setoodeh A , Saleh‐Gohari N , Astuti D , Barrett TG . Identification of homozygous WFS1 mutations (p.Asp211Asn, p.Gln486*) causing severe Wolfram syndrome and first report of male fertility. Eur J Hum Genet. 2013;21(3):347‐351. doi:10.1038/ejhg.2012.154 22781099 PMC3573194

[25] Kesavadev J , Kumar A , Shankar A , et al. An Asian Indian woman with Wolfram syndrome on insulin pump: successful pregnancy and beyond. Diabetes Technol Ther. 2011;13(7):781‐785. doi:10.1089/dia.2010.0242 21517693

[26] Rugolo S , Mirabella D , Palumbo MA , Chiantello R , Fiore G . Complete Wolfram's syndrome and successful pregnancy. Eur J Obstet Gynecol Reprod Biol. 2002;105(2):192‐193. doi:10.1016/s0301-2115(02)00150-1 12381487

[27] Chaussenot A , Bannwarth S , Rouzier C , et al. Neurologic features and genotype‐phenotype correlation in Wolfram syndrome. Ann Neurol. 2011;69(3):501‐508. doi:10.1002/ana.22160 21446023

[28] Richards S , Aziz N , Bale S , et al. Standards and guidelines for the interpretation of sequence variants: a joint consensus recommendation of the American College of Medical Genetics and Genomics and the Association for Molecular Pathology. Genet Med. 2015;17(5):405‐424. doi:10.1038/gim.2015.30 25741868 PMC4544753

