# Supplementary figures and images for: Gonadal function in males with WFS1 spectrum disorder (Wolfram syndrome)—A European cohort perspective
Source: Andrology. 2025 Apr 29;14(2):398–410. doi: 10.1111/andr.70049 (PMC12842843; doi:10.1111/andr.70049)

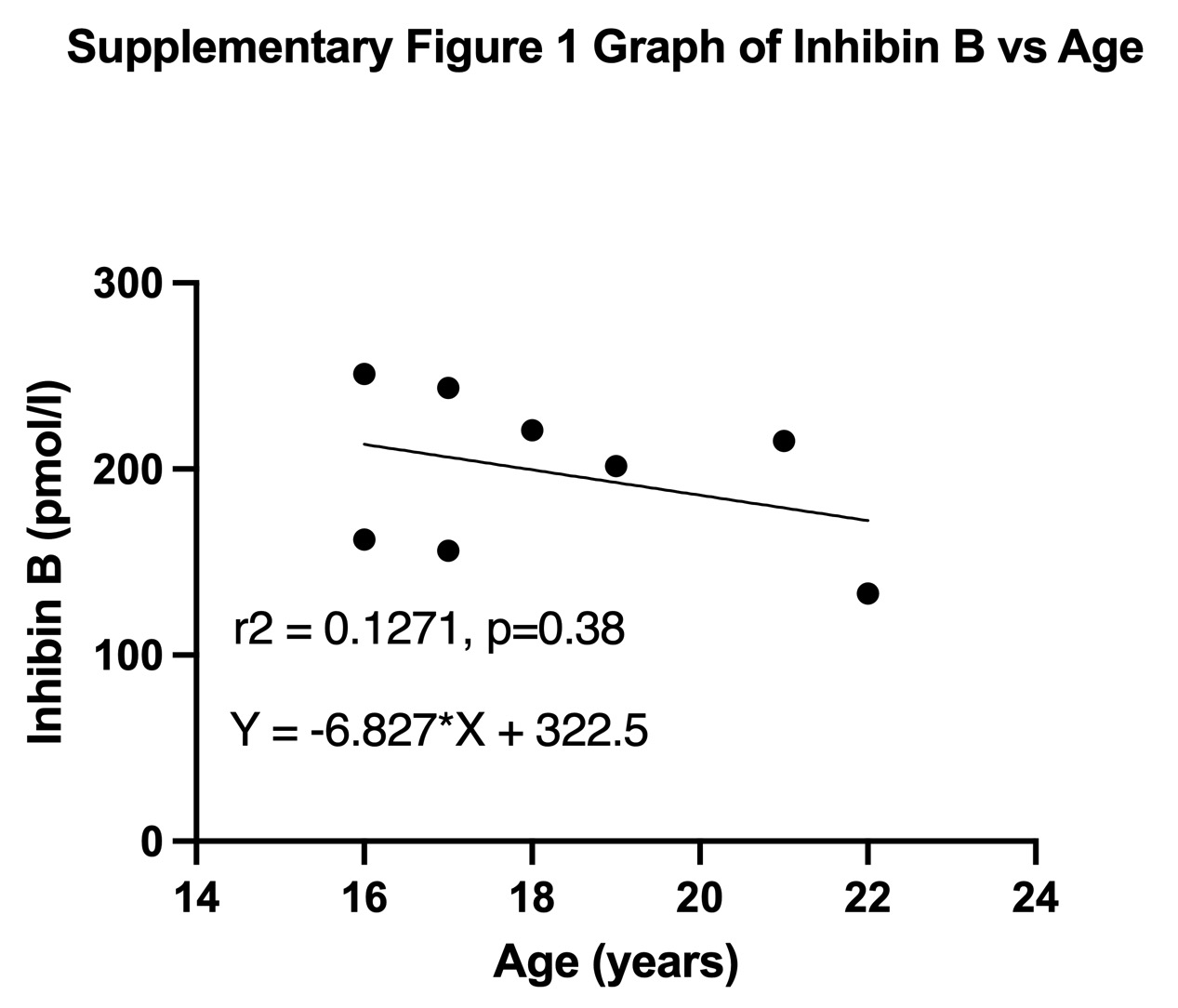

Supplement: Supplementary file 1 — Supporting Information [file ANDR-14-398-s001.jpg]
